# Comparison of craniofacial skeletal morphology in pediatric obstructive sleep apnea-hypopnea syndrome patients with class II and class III malocclusions: a retrospective cross-sectional study

**DOI:** 10.3389/fped.2026.1682284

**Published:** 2026-03-05

**Authors:** Lijia Deng, Hongjie Song

**Affiliations:** Department of Stomatology, Chengdu Second People’s Hospital, Chengdu, China

**Keywords:** class III malocclusion, craniofacial, malocclusions, obstructive sleep apnea-hypopnea syndrome, skeletalpatterns

## Abstract

**Introduction:**

This study aimed to investigate craniofacial skeletal morphology in pediatric obstructive sleep apnea-hypopnea syndrome (OSAHS) patients with different malocclusion types.

**Materials and methods:**

A retrospective cross-sectional analysis study was conducted at Chengdu Second People's Hospital from 2023 to 2024. A total of 299 children diagnosed with OSAHS (aged 10–12 years) were included. Craniofacial structures were assessed using Jarabak and Ricketts methods. Patients were divided by malocclusion type: Class II (*n* = 150, 56.7% male, mean age 11.2 ± 0.6) and Class III (*n* = 149, 50.3% male, mean age 11.4 ± 0.8). Group differences in cephalometric parameters were compared using independent samples t-tests and Mann–Whitney U tests, as appropriate.

**Results:**

Significant differences were found between groups. Compared to Class II, Class III patients had lower ANB angle (−2.8 ± 2.6° vs. 5.6 ± 1.9°, Cohen's d = 1.23), Wits appraisal (–2.9 ± 3.7 mm vs. 2.6 ± 0.7 mm, Cohen's d = 2.02), SN:GoMe (103.1 ± 6.0% vs. 106.7 ± 9.0%, Cohen's d = 0.48), MP/FH (32.9 ± 2.99° vs. 37.3 ± 2.65°, Cohen's d = 1.57), Xi-Pm/DC-Xi (29.3 ± 2.1° vs. 24.6 ± 3.1°, Cohen's d = 1.81), and ANS-Xi-Pm (47.8 ± 2.9° vs. 50.7 ± 2.4°, Cohen's d = 1.11) (all *P* < 0.05). Class III patients showed higher S-Ar:Ar-Go (75.9 ± 7.2% vs. 80.9 ± 13.0%, Cohen's d = 0.48), NP/FH (88.1 ± 2.9° vs. 83.1 ± 1.86°, Cohen's d = 2.14), Pt-Gn/Ba-N (92.2 ± 2.1° vs. 79.6 ± 2.4°, Cohen's d = 5.44), and Hy-C3 distance (5.8 ± 0.9 mm vs. 3.4 ± 0.4 mm, Cohen's d = 3.26) (all *P* < 0.05). No significant differences were observed in other parameters (N-S-Ar, S-Ar-Go; *P* > 0.05).

**Conclusion:**

Distinct craniofacial skeletal patterns exist in pediatric OSAHS patients with different malocclusions. Class III patients demonstrate mandibular growth restriction with compensatory protrusion, while Class II patients display high-angle, long-face morphology with clockwise growth rotation. These findings have important clinical implications for individualized orthodontic and surgical planning in the management of pediatric OSAHS, highlighting the need for early assessment of craniofacial structure in affected children.

## Introduction

Obstructive sleep apnea-hypopnea syndrome (OSAHS) is a common pediatric sleep disorder, marked by recurrent upper airway obstruction during sleep and resulting in intermittent hypoxia and disrupted sleep architecture (1, 2). The prevalence of OSAHS in children is as high as 5.7%, and is particularly increased in those with risk factors such as obesity, tonsillar hypertrophy, or craniofacial skeletal anomalies (3). The pathophysiology of pediatric OSAHS involves a complex interplay between anatomical and neuromuscular factors, including increased neck soft tissue, impaired airway muscle tone, and craniofacial skeletal morphology, all contributing to upper airway collapsibility (4, 5).

Malocclusion, a prevalent dental condition affecting over 60% of the global population (6), has been identified as a significant risk factor for OSAHS. Skeletal patterns are commonly classified according to the ANB angle, which reflects the sagittal relationship between the maxilla and mandible, as well as by other cephalometric parameters. Class II skeletal pattern is typically characterized by maxillary protrusion and/or mandibular retrusion, whereas Class III pattern involves maxillary hypoplasia, mandibular prognathism, or both (6, 7). In addition to the ANB angle, parameters such as the SNA angle (indicating the anteroposterior position of the maxilla relative to the cranial base) and the facial depth angle (Ricketts), which assesses the position of the mandible, are essential for a comprehensive evaluation of craniofacial skeletal morphology (8). Importantly, a normal ANB angle does not always exclude skeletal discrepancies; for example, biretrusive skeletal patterns can be present with reduced SNA and facial depth angles despite a normal ANB, still resulting in reduced oropharyngeal airway space (9).

Craniofacial skeletal anomalies, such as retrognathia or midface hypoplasia, may contribute to upper airway narrowing and increase susceptibility to OSAHS (10). Furthermore, the relationship between OSAHS and craniofacial development is bidirectional: chronic upper airway obstruction can promote mouth breathing, disrupt nasal function, alter craniofacial muscle balance, and ultimately impair normal skeletal growth (11). This cycle may exacerbate skeletal deformities, further compromising airway patency. Early recognition and intervention targeting craniofacial anomalies have the potential to improve respiratory outcomes and quality of life in affected children (12, 13).

Despite growing recognition of the association between craniofacial skeletal morphology and pediatric OSAHS, there is a notable lack of comparative data specifically evaluating craniofacial characteristics between Class II and Class III malocclusion in OSAHS patients. Understanding these differences is critical, as they may inform individualized orthodontic or surgical treatment strategies (4).

Therefore, this study aims to compare the craniofacial skeletal morphology of pediatric OSAHS patients with Class II and Class III malocclusions, using a comprehensive set of cephalometric parameters. Our findings are intended to provide evidence to support clinical decision-making and the development of tailored management plans for children with OSAHS and varying skeletal patterns.

## Materials and methods

### Study design and participant

This retrospective, single-center study included pediatric OSAHS patients treated at the Department of Stomatology, Chengdu Second People's Hospital, between January 2023 and December 2024. The study was approved by the Ethics Committee of Chengdu Second People's Hospital (Approval No. 2023172), and informed consent was obtained from the guardians of the participants.

The inclusion criteria were: (1) All pediatric patients were diagnosed with OSAHS according to established diagnostic criteria (14, 15), confirmed by lateral cephalometric radiographs and overnight polysomnography (PSG) performed using a standardized protocol (Alice 6, Philips Respironics). The PSG included EEG, EOG, EMG, ECG, airflow (nasal pressure transducer and thermistor), respiratory effort (thoracic and abdominal bands), oxygen saturation, and snoring sensors. OSAHS was diagnosed if the apnea-hypopnea index (AHI) exceeded 1 event/hour, as per international pediatric guidelines. (2) Age range of 10–12 years and (3) Complete clinical data available. Exclusion criteria included: (1) a history of adenoidectomy or tonsillectomy; (2) genetic abnormalities; (3) previous orthodontic treatment; (4) craniofacial deformities, such as cleft lip and palate.

### Data collection

Medical records of all pediatric patients were retrieved from the electronic medical record system of Chengdu Second People's Hospital. Before treatment, lateral cephalometric radiographs were obtained using the CRANEX DR digital x-ray machine in the natural head position. Centric occlusion was ensured by instructing the patient to bite in maximum intercuspation of the posterior teeth, with lips relaxed and facial muscles at rest, under direct supervision by an experienced technician. Cephalometric analysis was conducted by experienced researchers using Dolphin-3D software (version 11.7; Dolphin Imaging, Chatsworth, CA, USA).

**Figure 1:**
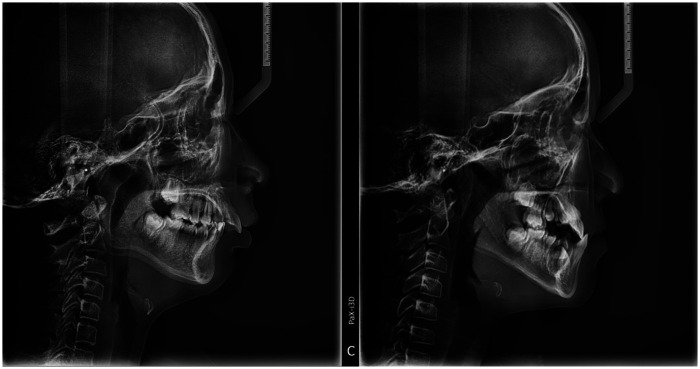
Typical images of class II and class III pediatric patients.

Patients were grouped according to established cephalometric norms: Class II skeletal pattern (ANB angle >2°) and Class III skeletal pattern (ANB angle <0°), reflecting maxillary protrusion/mandibular retrusion and mandibular prognathism/maxillary deficiency, respectively (16, 17). Patients with ANB values between 0° and 2° (skeletal Class I or borderline) were excluded. Typical images of Class II and Class III skeletal patterns are shown in Figure 1.

### Cephalometric landmarks and measurement methods

**Figure 2:**
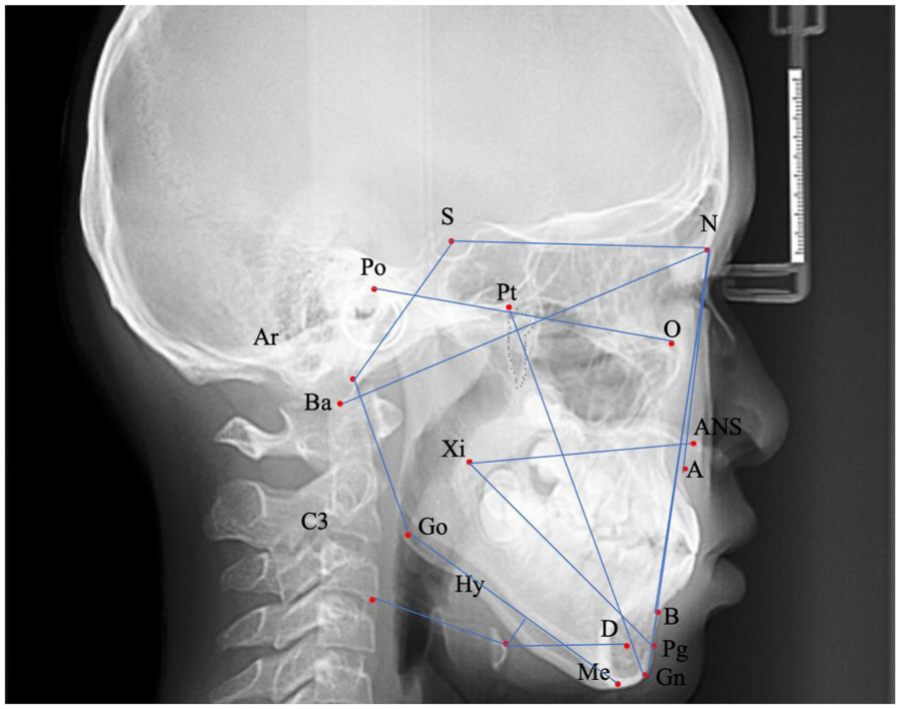
Cephalometric landmarks and reference points used in this study. Lateral cephalometric radiograph demonstrating all anatomical landmarks employed for measurement and analysis. Key landmarks are labeled as follows: N, nasion; S, sella; ANS, anterior nasal spine; A, subspinale (Point A); B, supramentale (Point B); Ar, articulare; Ba, basion; Pt, pterygomaxillary fissure; Po, porion; O, orbitale; Xi, xi point (mid-ramus); Go, gonion; Hy, hyoid bone; D, point D (mandibular landmark); Me, menton; Pg, pogonion; Gn, gnathion; C3, third cervical vertebra. Blue lines connect the key points according to measurement protocols. These landmarks correspond to the definitions and measurement items described in Table 1.

Cephalometric data from both groups were analyzed with emphasis on hyoid bone position. To ensure measurement accuracy, the primary examiner (T.Z.) completed structured calibration consisting of standardized training in cephalometric landmark identification, followed by practice on 20 external cephalograms until stable landmark placement was achieved. To reduce bias, formal measurements were performed twice by the same calibrated examiner with 2-week separation, using blinded, independent landmark re-identification rather than referring to prior records. Measurement reliability was evaluated using Dahlberg's formula: $E=\sqrt{\sum \frac{d^{2}}{2n}}$, for random error and the intraclass correlation coefficient (ICC). ICC was calculated using a 2-way mixed-effects model with absolute agreement to quantify repeatability after calibration. Linear and angular errors were summarized as absolute differences, and averaged values were used for analysis. Reliability met excellent standards: random error was <0.67 mm (linear) and <0.58° (angular), and ICC ranged 0.92–0.98 across all parameters, confirming strong intra-examiner consistency post-calibration and supporting measurement reproducibility beyond solely reporting error estimates. A comprehensive overview of all cephalometric landmarks and measurements—including points A, B, the ANB angle, and additional key anatomical reference points (such as N, S, ANS, Ba, Ar, Pt, Po, O, Xi, Go, Hy, D, Me, Pg, Gn, and C3), is illustrated in Figure 2. Each landmark used in the analysis is clearly identified in the figure, with corresponding definitions detailed in Table 1.

**Table 1:** Cephalometric items and definitions.

Items	Definition
SNA	The angle formed by lines between the sella, nasion, and subspinale points, which represents the sagittal position of the maxilla
SNB	The angle formed by lines between the sella, nasion, and supramental points, which represents the sagittal posit
ANB	The angle formed by lines between the subspinale, nasion, and supramental points, which represents the sagittal position of the maxilla and mandible
Wits	Draw perpendicular lines from the upper and lower alveolar ridge points A and B to the functional occlusal plane respectively. The two perpendicular feet are points Ao and Bo respectively. The distance between point Ao and point Bo measured is the Wits value
N-S-Ar	The NSAr angle is an important angle in orthodontic cephalometric analysis, which is used to evaluate the inclination of the mandibular ramus. It is the angle between the line connecting the sella turcica point (S) and the nasion point (N), and the line connecting the sella turcica point (S) and the articulare point (Ar)
S-Ar-Go	S-Ar-Go refers to the length of the line connecting from the sella turcica point (S) through the articulare point (Ar) to the gonion point (Go). It is a way to represent the length of the mandibl
Ar-Go-Me	Ar—Go—Me refers to the length of the line connecting from the articulare point (Ar) through the gonion point (Go) to the menton point (Me). This index is mainly used to evaluate the shape and growth of the mandible
N-Go-Ar	The N-Go-Ar angle refers to the angle between the line connecting the nasion point (N) and the gonion point (Go), and the line connecting the gonion point (Go) and the articulare point (Ar). In cephalometric analysis, the value of the N-Go-Ar angle is obtained by determining the positions of these three points on the cephalometric radiograph and then measuring the angle formed by the two lines.
N-Go-Me	The N—Go—Me refers to the angle formed by the line connecting the nasion point (N) and the gonion point (Go), and the line connecting the gonion point (Go) and the menton point (Me), which reflects the vertical growth of the mandible and the vertical proportion of the face.
SUM	Sum of the saddle and articular angles and mandibular angles, which can be used to evaluate and predict the growth direction of the face
S‒N/Go-Me	Ratio of the length from the anterior skull base to the mandible
S-Ar/Ar-Go	Ratio between the posterior skull base and the ascending mandibular
MP/FH	The angle between the plane of mandible inclination (MP) and the Frankfort horizontal (FH) plane, which indicates the angle of the mandible
NP/FH	NP/FH refers to the angle between the nasion-pterygomaxillary fissure line and the Frankfort horizontal plane
Pt-Gn/Ba-N	The ratio of the length of Pt-Gn to the length of Ba-N
Xi-Pm/DC-Xi	The ratio of the length of Xi—Pm to the length of DC—Xi
ANS-Xi-Pm	It represents the angle between the line connecting ANS and Xi and the line connecting Xi and Pm. This angle has some reference value for assessing the morphology, position, and anteroposterior development of the maxilla
S-Go/N-Me	Ratio between the posterior skull base and the ascending mandibular
Hy-C3	The shortest horizontal distance between the most anterior-inferior point on the body of the hyoid bone and the anterior border of the third cervical vertebra (C3)
Hy-Mp	The angle between the plane of mandible inclination (MP) and the Frankfort horizontal (FH) plane, which indicates the angle of the mandible
Hy-D	Linear distance between the hyoid and the mandibular D point

### Quantitative assessment of adenoid and tonsil hypertrophy

Adenoid Measurement: The maximum thickness of the adenoid and nasopharyngeal airway width were measured as follows: a perpendicular line (Ba) was drawn from the most prominent point of the adenoid inferior margin (Ad) to the tangent line of the clivus extracranial inclined plane (18). The intersection points with Ba and the soft palate were defined as D and Na, respectively. The Ad-D distance represented the adenoid maximum thickness, and the D-Na distance represented the airway width.

**Figure 3:**
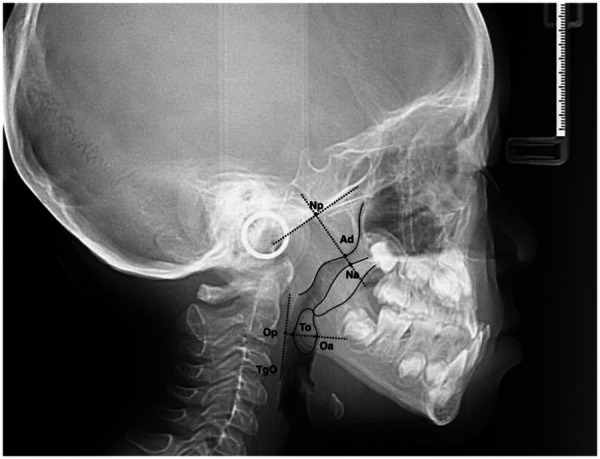
Landmarks and items for measurement.

Tonsil Measurement: Hypertrophic tonsils, manifested as elliptical shadows between the oropharynx and tongue base, were measured by drawing a tangent line along the posterior pharyngeal wall (19). A perpendicular line from the tonsil's most prominent point (To) to this tangent line yielded intersection point Op; its extension intersecting the tongue base was Oa. The Oa-To distance was the tonsil maximum thickness, and the Oa-Op distance was the oropharyngeal airway width (Figure 3).

#### Obstruction rate calculation

Adenoid and tonsil obstruction rates were calculated using the formulas: A/N × 100 and TS/TANS × 100, respectively. Obstruction was defined as adenoid size >50% of nasopharyngeal width or tonsil size >50% of oropharyngeal airway width; no obstruction was defined as ≤50% of the corresponding airway width.

### Statistical analysis

Statistical analyses were performed using SPSS 26.0 software (IBM, Armonk, NY, USA). Prior to hypothesis testing, missing data were assessed by calculating the missing proportion for each variable. Variables with missing rate <5% were considered to have low missingness and were imputed using multiple imputation (MI) with 5 datasets generated; continuous variables were imputed according to their distribution (mean for normal, median for skewed), and categorical variables were imputed using mode. For variables with missing rate ≥5%, the missingness mechanism was reviewed; If missingness was judged random, MI was applied, whereas cases with systematic missingness were excluded with reasons explicitly documented. After missing-data handling, continuous variables were presented as mean ± SD if normally distributed with equal variances (independent t-test) or as median (IQR) if skewed (Mann–Whitney *U*). Categorical variables were expressed as *n* (%) and analyzed using *χ*^2^ or Fisher's exact test when any expected cell count was < 5. Pearson correlation analyses between A/N and TS/TANS ratios and the AHI, as well as lowest oxygen saturation (LSaO_2_), were performed to assess the relationship between adenotonsillar hypertrophy (quantified by cephalometric measurements) and OSAHS severity. A two-sided *P* < 0.017 (Bonferroni-corrected) or *P* < 0.05 (single uncorrected test) indicated statistical significance.

## Results

### Demographic characteristics

A total of 385 pediatric records were retrieved. After excluding 86 cases for reasons such as a history of orthodontic treatment, incomplete data, or the absence of significant adenotonsillar hypertrophy, 299 participants who met all inclusion criteria were included in the final analysis. As per the study's inclusion criteria, all 299 participants (100%) had a confirmed diagnosis of adenoidal and/or tonsillar hypertrophy. Participants were categorized into Class II (*n* = 150) and Class III (*n* = 149) skeletal patterns. The two groups were comparable in sex distribution (56.7% vs. 50.3% male, respectively; *χ*^2^ = 0.92, *P* = 0.338). A minor but statistically significant age difference was noted, with the Class II group being slightly younger than the Class III group (11.2 ± 0.6 vs. 11.4 ± 0.8 years; mean difference: 0.2 years, *P* = 0.049). However, the effect size for this difference was small (Cohen's d = 0.28, 95% CI: −0.01 to 0.57) (Table 2).

**Table 2:** Cephalometric landmarks and measurement methods.

Variable	Class II (*n* = 150)	Class III (*n* = 149)	Cohen's d, 95% CI	*P*
Sex, male	85 (56.7%)	75 (50.3%)	0.056, (0.00–0.14)	>0.05
Age, years	11.2 ± 0.6	11.4 ± 0.8	0.28, (−0.01–0.57)	>0.05

### Correlation of adenoid and tonsil hypertrophy with OSAHS severity

**Figure 4:**
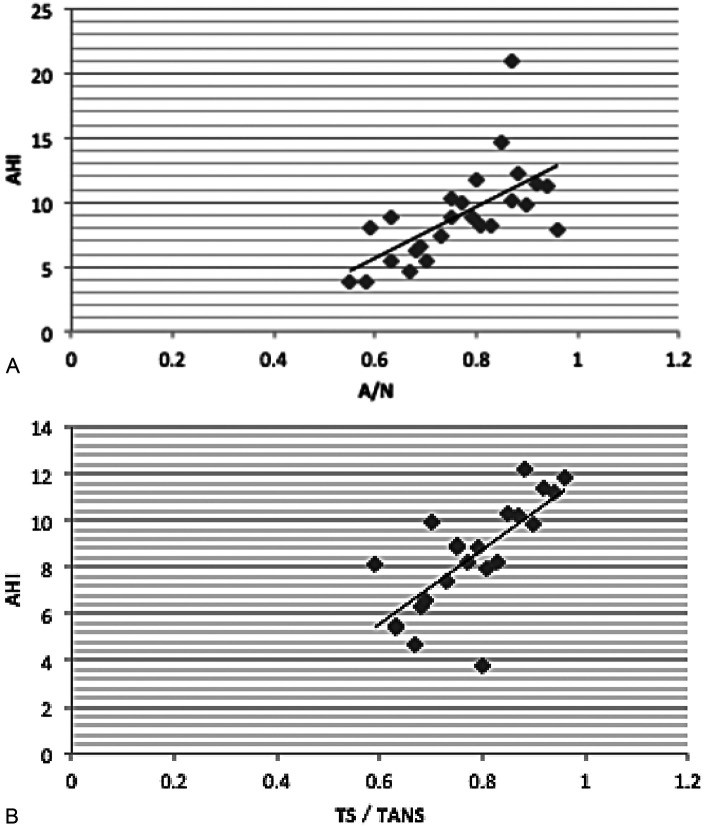
**(A)** scatter plot of A/N ratio vs. AHI Value (r = 0.644, P < 0.05). **(B)** Scatter Plot of TS/TANS Ratio vs. AHI Value (*r* = 0.673, *P* < 0.05).

To investigate the relationship between adenotonsillar hypertrophy and sleep-disordered breathing, we analyzed correlations between cephalometric and polysomnographic data. The analysis revealed a significant positive correlation between the A/N ratio and AHI in the adenoid hypertrophy group (*r* = 0.644, *P* < 0.05, Figure 4A), and similarly between the TS/TANS ratio and AHI in the tonsillar hypertrophy group (*r* = 0.673, *P* < 0.05, Figure 4B). Conversely, no significant correlation was found between these anatomical ratios (A/N and TS/TANS) and LSaO_2_ (Table 3). Paired t-test analysis also confirmed no significant differences in AHI or LSaO_2_ between the two groups (*P* > 0.05 for both, Table 4).

**Table 3:** Weak correlations between airway ratios and LSaO₂ in the adenoid hypertrophy group and tonsillar hypertrophy group.

Group	Ratio	LSaO2	R
Adenoid	0.76 ± 0.09	0.89 ± 0.03	0.388
Tonsil	0.79 ± 0.10	0.90 ± 0.10	0.112

**Table 4:** No significant differences in AHI and LSaO₂ between the Two groups (*P* > 0.05).

Index	Adenoid	Tonsil	*P*
AHI	8.98 ± 3.67	8.09 ± 2.46	0.08
LSaO2	0.89 ± 0.03	0.90 ± 0.04	0.77

### Cephalometric analysis of class II and class III group

The Class III group showed a smaller ANB angle (−2.8° ± 2.6°) than the Class II group (5.6° ± 1.9°), mean difference −2.8° (Cohen's d = 1.23, 95% CI 0.96–1.50; *P* < 0.001). Wits appraisal was lower in Class III (–2.9 ± 3.7 mm) than Class II (2.6 ± 0.7 mm), mean difference −5.5 mm (Cohen's d = 2.02, 95% CI 1.70–2.34; *P* < 0.001). The SN:GoMe percentage was lower in Class III (103.1% ± 6.0%) than Class II (106.7% ± 9.0%), mean difference −3.6% (Cohen's d = 0.48, 95% CI 0.19–0.77; *P* = 0.039). The S-Ar:Ar-Go percentage was 75.9% ± 7.2% in Class III and 80.9% ± 13.0% in Class II, mean difference −5.0% (Cohen's d = 0.48, 95% CI 0.17–0.79; *P* = 0.027).

Mandibular plane orientation differed with a large effect: MP/FH was smaller in Class III (32.9° ± 2.99°) than Class II (37.3° ± 2.65°), mean difference −4.4° (Cohen's d = 1.57, 95% CI 1.26–1.88; *P* < 0.001). The NP/FH angle was higher in Class III (88.1° ± 2.9°) than Class II (83.1° ± 1.86°), mean difference 5.0° (Cohen's d = 2.14, 95% CI 1.89–2.39; *P* < 0.001). The Pt-Gn/Ba-N angle showed very strong separation: 92.2° ± 2.1° in Class III vs. 79.6° ± 2.4° in Class II, mean difference 12.6° (Cohen's d = 5.44, 95% CI 4.89–5.99; *P* < 0.001). The Xi-Pm/DC-Xi angle was 29.3° ± 2.1° in Class III vs. 24.6° ± 3.1° in Class II, mean difference 4.7° (Cohen's d = 1.81, 95% CI 1.49–2.13; *P* < 0.001). The ANS-Xi-Pm angle was smaller in Class III (47.8° ± 2.9°) than Class II (50.7° ± 2.4°), mean difference −2.9° (Cohen's d = 1.11, 95% CI 0.84–1.38; *P* < 0.001). Hyoid position differed markedly: the Hy-C3 distance was larger in Class III (5.8 ± 0.9 mm) than Class II (3.4 ± 0.4 mm), mean difference 2.4 mm (Cohen's d = 3.26, 95% CI 2.88–3.64; *P* < 0.001). Other cephalometric measurements (N-S-Ar, S-Ar-Go, Ar-Go-Me, N-Go-Ar, N-Go-Me, SUM, S-Go:N-Me, Hy-MP, Hy-D) showed no significant differences (*P* > 0.05). Full numerical results are presented in Table 5.

**Table 5:** Comparison of the cephalometric results between the two groups.

Cephalometric Items	Class II (*n* = 150)	Class III (*n* = 149)	Cohen's d, 95% CI	*P*
ANB (°)	5.6 ± 1.9	−2.8 ± 2.6	**1.23,** (**0.96–1.50)**	**<0**.**001**
Wits (mm)	2.6 ± 0.7	−2.9 ± 3.7	**2.02,** (**1.70–2.34)**	**<0**.**001**
N-S-Ar (°)	123.3 ± 2.90	122.7 ± 2.09		0.646
S-Ar-Go (°)	150.2 ± 2.21	150.9 ± 2.90		0.711
Ar-Go-Me (°)	123.2 ± 2.93	123.6 ± 3.90		0.784
N-Go-Ar (°)	48.9 ± 1.95	49.50 ± 1.72		0.675
N-Go-Me (°)	74.3 ± 2.73	75.3 ± 2.28		0.389
SUM (°)	404.7 ± 2.65	403.3 ± 3.56		0.504
SN: GoMe (%)	106.7 ± 9.0	103.1 ± 6.0	**0.48,** (**0.19–0.77)**	**0**.**039**
S-Ar: Ar-Go (%)	80.9 ± 13.0	75.9 ± 7.2	**0.48,** (**0.17–0.79)**	**0**.**027**
MP/FH (°)	37.3 ± 2.65	32.9 ± 2.99	**1.57,** (**1.26–1.88)**	**<0**.**001**
NP/FH (°)	83.1 ± 1.86	88.1 ± 2.9	**2.14,** (**1.89–2.39)**	**<0**.**001**
S-Go:N-Me (%)	63.2 ± 4.1	62.6 ± 3.9		0.512
Pt-Gn/Ba-N (°)	79.6 ± 2.4	92.2 ± 2.1	**5.44,** (**4.89–5.99)**	**<0**.**001**
Xi-Pm/DC-Xi (°)	24.6 ± 3.1	29.3 ± 2.1	**1.81,** (**1.49–2.13)**	**<0**.**001**
ANS-Xi_Pm (°)	50.7 ± 2.4	47.8 ± 2.9	**1.11,** (**0.84–1.38)**	**<0**.**001**
Hy-C3 (mm)	3.4 ± 0.4	5.8 ± 0.9	**3.26,** (**2.88–3.64)**	**<0**.**001**
Hy-MP (mm)	1.8 ± 0.9	1.7 ± 0.8		0.639
Hy-D (mm)	6.2 ± 1.3	6.3 ± 1.4		0.478

Bold values indicate statistically significant differences between Class II and Class III groups (*P* < 0.05).

The largest effect magnitudes in measurements mechanistically linked to airway obstruction or OSAHS severity were observed for Hy-C3 (mean difference 2.4 mm; Cohen's d = 3.26, 95% CI 2.88–3.64; *P* < 0.001) and MP/FH (mean difference −4.4°; Cohen's d = 1.57, 95% CI 1.26–1.88; *P* < 0.001). Greater Hy-C3 values reflect a more anteriorly positioned hyoid relative to the cervical spine, while steeper mandibular plane inclination (higher MP/FH) characterizes the Class II group. Both metrics demonstrated strong statistical separation and represent structural features relevant to retroglossal and hypopharyngeal airway dimensions. The magnitude and direction of between-group contrasts indicate that airway-related skeletal variation is centered on hyoid position and mandibular orientation, rather than global craniofacial proportions, supporting their relevance as anatomical markers associated with airway patency and OSAHS phenotypic differences.

## Discussion

This study examined the craniofacial skeletal morphology of pediatric OSAHS patients with different malocclusions. This study is to critically interpret and contextualize these findings within current clinical and scientific understanding, while drawing out their implications for orthodontic and surgical management.

The shape of the upper airway is affected by the size, shape, and location of the skull, upper mandible, and hyoid. Conversely, an abnormal upper airway can also affect craniofacial development (20). The development of the maxillofacial region from birth to the end of growth has four growth peaks. The second peak of maxillofacial development in children occurs at the age of 4–7 years, coinciding with peak tonsil and adenoid proliferation. These two independent continuous growth processes may cause airway obstruction once there is an imbalance. A previous study reported that obstruction of any part of the upper airway could cause facial abnormalities in children (21–23). As early diagnosis and treatment of skeletal anomalies before puberty can enhance treatment success and reduce treatment duration (24), this study focuses on the relationship between skeletal anomalies and OSAHS in school-aged children.

During the developmental process of children, the shape and function of the upper respiratory tract are crucial for normal craniofacial development (25). Obstruction of the upper respiratory tract, especially during critical growth stages in children, can lead to craniofacial developmental abnormalities (26). OSAHS is a common issue in children, often associated with hypertrophy of the tonsils and adenoids. This condition not only affects respiratory function but can also result in changes to the facial skeletal structure, subsequently impacting occlusal relationships and the development of the jaw and face (27, 28).

Critically, our findings indicate that the most pronounced skeletal discrepancies between Class II and Class III OSAHS patients—such as the ANB angle, Wits appraisal, and hyoid bone position—carry direct implications for both airway patency and the selection of orthodontic or surgical interventions. In particular, the anterior and superior positioning of the hyoid in Class III patients, and the relatively retruded mandible in Class II patients, suggest different mechanisms of airway compromise and thus potentially distinct clinical management pathways.

The results of this study indicate that significant differences in the ANB angle and Wits analysis values between Class II and Class III patients suggest a marked change in the relative positional relationship between the maxilla and mandible. This is consistent with previous literature, where many studies have confirmed that mandibular retrognathism or maxillary prognathism are the primary factors leading to Class III malocclusion (29). Class II patients exhibited a relatively shorter mandibular length, which aligns with the facial characteristics of Class II patients mentioned in the literature, typically presenting a shorter mandibular body and relatively higher facial vertical height (30, 31). In studies concerning the position and function of the hyoid bone, it plays a crucial role in the anatomical structure of the pharyngeal airway (32). This study found that the position of the hyoid bone in Class III patients shifted anteriorly and was closer to the mandible, consistent with prior research, indicating that the relationship between the hyoid and mandible may play a key role in the patency of the upper airway (33, 34). The distance between the hyoid and mandible mentioned in this study showed no significant differences between the two groups; however, combined with the analysis of the distance between the hyoid and the third cervical vertebra, it further confirmed the shorter mandibular length in Class II patients. This finding emphasizes the importance of mandibular development on airway morphology and resonates with existing research, suggesting that mandibular growth patterns may be influenced by hyoid position (35, 36). Furthermore, the observed gonial angles in both groups were within the normal range, indicating that the condylar and glenoid fossa positions were normal. This contradicts the viewpoint of Dr. Jarabak (37), who suggested that Class III patients should have a gonial angle less than 120°. However, our results showed that both groups exhibited larger joint angles and similar measurements, suggesting a deficiency in muscle position and strength in both groups, which may lead to increased leverage effects in the posterior dental area. The literature has noted that when the maxillary angle is less than 52°, the forward growth of the mandible will be restricted (38). In this study, the N-Go-Ar values for both groups were below this critical threshold, which aligns with the mandibular developmental characteristics of Class II malocclusion but is inconsistent with the mandibular growth characteristics of Class III patients (39). This suggests that there may be different biological mechanisms underlying the growth patterns of the mandible in Class II and Class III patients, warranting further investigation.

In addition to skeletal morphology, this study directly addressed the relationship between anatomical airway obstruction and the functional severity of OSAHS. Quantitative cephalometric analysis revealed that both the A/N ratio and the TS/TANS ratio were significantly elevated in our cohort, confirming the presence of upper airway obstruction by Fujioka and Baroni criteria. More importantly, we observed strong positive correlations between these cephalometric obstruction indices and the AHI measured by overnight polysomnography (*r* = 0.644 for A/N and *r* = 0.673 for TS/TANS, both *P* < 0.05). This finding demonstrates that the degree of nasopharyngeal and oropharyngeal narrowing, as measured on standardized lateral cephalograms, is closely associated with the clinical severity of OSAHS in children.

These results support previous studies linking adenotonsillar hypertrophy to OSAHS, (13) but also expand on them by providing objective, quantifiable metrics that may be easily incorporated into routine orthodontic and sleep medicine practice. While some prior reports have questioned the reliability of two-dimensional cephalometric airway assessment due to inherent anatomical and technical variability (40), our use of strict image acquisition protocols and examiner calibration minimized bias and ensured reproducibility. Furthermore, despite the limitations of 2D imaging in capturing airway volume, our data suggest that linear surrogates (A/N and TS/TANS ratios) remain clinically meaningful, especially when combined with PSG-derived functional endpoints.

Interestingly, although the A/N and TS/TANS ratios were strongly correlated with AHI, their relationship with the LSaO_2_ was weak, suggesting that anatomic narrowing may be a more sensitive predictor of apnea frequency than of hypoxemia severity—potentially due to the rapid respiratory rates and compensatory mechanisms in pediatric patients (41). Nonetheless, mean LSaO_2_ values below 90% in both groups confirm the presence of clinically relevant hypoxemia, underscoring the need for early recognition and intervention.

Our findings have several clinical implications. First, cephalometric evaluation of adenoids and tonsils can serve as a practical screening tool for identifying children at greatest risk for moderate-to-severe OSAHS, particularly in resource-limited settings where access to PSG is restricted. Second, the integration of airway and skeletal assessment supports a more personalized approach to orthodontic and surgical management, with interventions tailored not only to correct malocclusion but also to optimize airway patency. For example, early orthopedic management in Class II patients with significant mandibular retrusion and airway narrowing may prevent progressive craniofacial and respiratory sequelae. In contrast, Class III patients may benefit from targeted maxillary advancement or combined surgical approaches that consider both skeletal and soft tissue contributions to airway obstruction. Contrary to some prior studies suggesting isolated tonsillar hypertrophy is not an independent risk factor for OSAHS (42), our results highlight a strong correlation between both adenoidal and tonsillar hypertrophy and the functional severity of pediatric OSAHS. This reinforces the importance of thorough upper airway evaluation in all children with suspected sleep-disordered breathing, regardless of the predominant pattern of lymphoid tissue enlargement. Our findings highlight the clinical importance of early interception and correction of craniofacial dysmorphosis during pediatric development, with particular attention to the preservation and improvement of oropharyngeal airway volume.”

Our study also raises new clinical hypotheses: for example, whether early orthopedic intervention in Class II OSAHS patients could prevent secondary airway and craniofacial sequelae, or whether postoperative changes in hyoid position after bimaxillary or chin surgery directly modulate OSAHS severity. Prospective studies and long-term follow-up are needed to address these questions.

Limitations of this study include its retrospective design, which inherently limits the ability to establish causal relationships and control for potential confounding factors. Although potential confounders such as body mass index (BMI) and adenotonsillar hypertrophy were extracted from medical records when available, these variables were not systematically recorded or statistically adjusted in the analysis. As a result, residual confounding may exist, and the observed skeletal differences cannot be attributed solely to malocclusion type. Additionally, because the study did not conduct a formal *a priori* sample size calculation, the sample size was determined by the number of complete and eligible cases available in the medical records. This may limit the statistical power to identify small skeletal differences, and some nonsignificant results could reflect limited sample size rather than a true absence of group differences. The single-center design may introduce selection bias and limit generalizability to other populations or ethnic groups. Variations in imaging quality, diagnostic protocols, and examiner calibration—even though minimized—are also inherent to retrospective studies and could affect measurement reliability. Furthermore, our study did not evaluate the transverse dimension of the upper jaw, particularly maxillary width, as we did not obtain three-dimensional imaging (such as CT or CBCT) due to concerns about radiation exposure in the pediatric population. This precluded a detailed assessment of maxillary transversality and its relationship to airway resistance, especially in Class III skeletal patterns. Future research utilizing three-dimensional imaging modalities will be essential to clarify the role of maxillary width and the effects of palatal expansion on airway function in pediatric OSAHS. Future research should also prioritize prospective, multicenter cohort studies with comprehensive assessment of confounders and longitudinal follow-up to clarify causal relationships between craniofacial development and OSAHS. Randomized trials of functional appliances or surgical interventions, with airway and skeletal outcomes as primary endpoints, would be particularly valuable. Integration of three-dimensional imaging and dynamic airway assessment may also provide deeper insights into the interplay between craniofacial structure and airway function in pediatric OSAHS.

## Conclusion

This study elucidates the craniofacial skeletal characteristics of pediatric OSAHS patients across different malocclusion patterns. Specifically, among Class III children with OSAHS, we observed features suggesting that although the mandible is relatively short overall (indicating growth restriction), it is positioned more forward (protruded) as a compensatory adaptation to maintain airway patency. This forward positioning attempts to reduce airway obstruction, but results in a more pronounced Class III malocclusion. In contrast, Class II children display a high-angle, long-face morphology, characterized by a larger gonial angle, shorter mandibular ramus, and clockwise growth rotation, indicating adaptive changes associated with OSAHS. These findings underscore the complex interplay between sleep-disordered breathing and craniofacial development. Therefore, in the management of pediatric craniofacial dysmorphosis, it is essential to consider oropharyngeal airway volume as a key factor in both diagnosis and treatment planning.

## Data Availability

The original contributions presented in the study are included in the article/Supplementary Material, further inquiries can be directed to the corresponding author/s.

## References

[1] Li Z Cai S Qiao J Li Y Wang Q Chen R. Implications of depressive mood in OSAHS patients: insights from event-related potential. BMC Psychiatry. (2024) 24:307. 10.1186/s12888-024-05772-6 38654234 PMC11040885

[2] Mao Z Zheng P Zhu X Wang L Zhang F Liu H Obstructive sleep apnea hypopnea syndrome and vascular lesions: an update on what we currently know. Sleep Med. (2024) 119:296–311. 10.1016/j.sleep.2024.05.010 38723575

[3] Picherit M Trentesaux T Ternisien A Foumou N Delfosse C Marquillier T. Management of obstructive sleep apnea-hypopnea syndrome in children: what is the role of orthodontics? A scoping review. Sleep Breath. (2025) 29:127. 10.1007/s11325-025-03288-1 40080307 PMC11906523

[4] Liu Y Zhou JR Xie SQ Yang X Chen JL. The effects of orofacial myofunctional therapy on children with OSAHS’s craniomaxillofacial growth: a systematic review. Children (Basel). (2023) 10(4):15. 10.3390/children10040670 PMC10136844 37189919

[5] Paradowska-Stolarz AM. Is malocclusion a risk factor for obstructive sleep apnea and temporomandibular disorders? An orthodontic point of view. Dent Med Probl. (2025) 62:197–9. 10.17219/dmp/194232 40249025

[6] Mecenas P Bastos R Fagundes NCF Normando D. Precision wings treating skeletal class II in growing patients: a systematic review and meta-analysis. Prog Orthod. (2025) 26:16. 10.1186/s40510-025-00564-4 40415149 PMC12104119

[7] Yu T Lv D Xu D Wang H Xiong X Cheng Q. Camouflage treatment of severe skeletal class III malocclusion with effective torque control in an adolescent combined with forward functional shift and hypodivergent. BMC Oral Health. (2025) 25:762. 10.1186/s12903-025-06113-z 40405176 PMC12096536

[8] Camcı H Salmanpour F. Cephalometric evaluation of anterior cranial base slope in patients with skeletal class I malocclusion with low or high SNA and SNB angles. Turk J Orthod. (2020) 33:171–6. 10.5152/TurkJOrthod.2020.20017 32974063 PMC7491964

[9] Boccalari E Rossi O Baldini B Tripicchio C Serafin M Caprioglio A. The maxillomandibular sagittal assessment: the ABwise appraisal and its correlation with ANB angle. J Clin Med. (2025) 14(4):1379. 10.3390/jcm14041379 40004908 PMC11856202

[10] Liu Y Lun H Hu Q Wei L Ye L Zhu S. Dynamic behavior of the oropharynx airway during deep breath in patients with obstructive sleep apnoea hypopnoea syndrome observed by ultrasonography. Sci Rep. (2025) 15:5585. 10.1038/s41598-025-90312-9 39955426 PMC11829957

[11] Pan H Xie J Gao W Chen W. [Characteristics of flow and heat transfer in the upper respiratory tract of humans under nasal obstructive conditions]. Sichuan Da Xue Xue Bao Yi Xue Ban. (2025) 56:215–21. 10.12182/20250160102 40109458 PMC11913997

[12] Cao JJ Han ZG Zhang J. [Difference in lateral cephalogram of the male patients with uygur and han OSAHS]. Lin Chuang Er Bi Yan Hou Tou Jing Wai Ke Za Zhi. (2016) 30:474–7. 10.13201/j.issn.1001-1781.2016.06.014 29871043

[13] Alassiry AM. Accuracy of different cephalometric analyses in the diagnosis of class III malocclusion in Saudi and Yemeni population. J Orthod Sci. (2020) 9:14. 10.4103/jos.JOS_21_20 33354540 PMC7749450

[14] Mao X Zhao W. Efficacy of montelukast combined with sublingual immunotherapy in the treatment of children with obstructive sleep apnea hypopnea syndrome complicated with allergic rhinitis. Pak J Med Sci. (2023) 39:1350–4. 10.12669/pjms.39.5.6985 37680839 PMC10480714

[15] Lu Y Fu Q Cai X Shen Y Wu J Qiu H. The potential of tRF-21-U0EZY9X1B plasmatic level as a biomarker of children with obstructive sleep apnea-hypopnea syndrome. BMC Pediatr. (2023) 23:197. 10.1186/s12887-023-04020-2 37101156 PMC10134554

[16] Brito FC Brunetto DP Nojima MCG. Three-dimensional study of the upper airway in different skeletal class II malocclusion patterns. Angle Orthod. (2019) 89:93–101. 10.2319/112117-806.1 30230378 PMC8137119

[17] Fernandez CCA Pereira C Luiz RR Vieira AR De Castro Costa M. Dental anomalies in different growth and skeletal malocclusion patterns. Angle Orthod. (2018) 88:195–201. 10.2319/071917-482.1 29215300 PMC8312537

[18] Isono S Shimada A Utsugi M Konno A Nishino T. Comparison of static mechanical properties of the passive pharynx between normal children and children with sleep-disordered breathing. Am J Respir Crit Care Med. (1998) 157:1204–12. 10.1164/ajrccm.157.4.9702042 9563740

[19] Ozdemir H Söyüncü Y Ozgörgen M Dabak K. Effects of changes in heel fat pad thickness and elasticity on heel pain. J Am Podiatr Med Assoc. (2004) 94:47–52. 10.7547/87507315-94-1-47 14729991

[20] Dempsey JA Skatrud JB Jacques AJ Ewanowski SJ Woodson BT Hanson PR Anatomic determinants of sleep-disordered breathing across the spectrum of clinical and nonclinical male subjects. Chest. (2002) 122:840–51. 10.1378/chest.122.3.840 12226022

[21] Linder-Aronson S. Adenoids. Their effect on mode of breathing and nasal airflow and their relationship to characteristics of the facial skeleton and the denition. A biometric, rhino-manometric and cephalometro-radiographic study on children with and without adenoids. Acta Otolaryngol Suppl. (1970) 265:1–132. 5272140

[22] Holmberg H Linder-Aronson S. Cephalometric radiographs as a means of evaluating the capacity of the nasal and nasopharyngeal airway. Am J Orthod. (1979) 76:479–90. 10.1016/0002-9416(79)90252-5 292310

[23] Linder-Aronson S Woodside DG Lundström A. Mandibular growth direction following adenoidectomy. Am J Orthod. (1986) 89:273–84. 10.1016/0002-9416(86)90049-7 3515955

[24] Atasever Isler AA Hezenci Y Bulut M. Prevalence of orthodontic malocclusion in children aged 10–12: an epidemiological study. BMC Oral Health. (2025) 25:249. 10.1186/s12903-025-05650-x 39966826 PMC11834610

[25] Mitteroecker P Stansfield E. A model of developmental canalization, applied to human cranial form. PLoS Comput Biol. (2021) 17:e1008381. 10.1371/journal.pcbi.1008381 33591964 PMC7909690

[26] Wang H Qiao X Qi S Zhang X Li S. Effect of adenoid hypertrophy on the upper airway and craniomaxillofacial region. Transl Pediatr. (2021) 10:2563–72. 10.21037/tp-21-437 34765480 PMC8578754

[27] Lin C Huang Y Lin Q. The impact of tonsillectomy and/or adenoidectomy on cognitive function and brain structure in pediatric patients with OSAHS. Technol Health Care. (2025) 33:321–31. 10.3233/thc-241028 39302401

[28] Ma Y Xie L Wu W. The effects of adenoid hypertrophy and oral breathing on maxillofacial development: a review of the literature. J Clin Pediatr Dent. (2024) 48:1–6. 10.22514/jocpd.2024.001 38239150

[29] Singh H Srivastava D Kapoor P Sharma P Mishra S Chandra L Anterior maxillary distraction for cleft palate associated severe hypoplastic maxillary class III deformity during adolescence—a case report. Int Orthod. (2024) 22:100927. 10.1016/j.ortho.2024.100927 39426200

[30] D'Antò V Oliva G Rongo R Bucci R Martina S Franchi L Morphologic predictors of mandibular changes induced by sander’s bite jumping appliance. Orthod Craniofac Res. (2025) 28:67–74. 10.1111/ocr.12850 39180251 PMC11701945

[31] Verma SL Tikku T Khanna R Srivastava K Maurya RP Rai P. Correlation of mandibular third molar orientation and available retromolar space with arch length discrepancy in subjects with different growth pattern. Natl J Maxillofac Surg. (2024) 15:106–15. 10.4103/njms.njms_63_22 38690237 PMC11057591

[32] Shetty V SS Ragu K. Pharyngeal airway dimensions and hyoid bone position in children with class II malocclusion and sleep problems: a cross sectional study. J Oral Biol Craniofac Res. (2024) 14:830–5. 10.1016/j.jobcr.2024.11.001 39611180 PMC11602553

[33] Oh JC. Changes in the activation level of the floor of the mouth muscles during pressing and swallowing tasks according to the degree of tongue pressure. Dysphagia. (2024) 39:1125–34. 10.1007/s00455-024-10691-5 38466426

[34] Saka N Chotirungsan T Yoshihara M Pan CR Tsutsui Y Dewa N Functional involvement of the sternohyoid muscle during breathing and swallowing in rats. Am J Physiol Gastrointest Liver Physiol. (2024) 327:G598–607. 10.1152/ajpgi.00138.2024 39104324

[35] Yue Z Yi Z Liu X Chen M Yin S Liu Q Comparison of invisalign mandibular advancement and twin-block on upper airway and hyoid bone position improvements for skeletal class II children: a retrospective study. BMC Oral Health. (2023) 23:661. 10.1186/s12903-023-03295-2 37705022 PMC10500932

[36] Liu Y Chen W Wei Y Zhang G Zhang X Sharhan HM The effect of orthodontic vertical control on the changes in the upper airway size and tongue and hyoid position in adult patients with hyperdivergent skeletal class II. BMC Oral Health. (2022) 22:532. 10.1186/s12903-022-02580-w 36424588 PMC9686087

[37] Rodriguez-Cardenas YA Arriola-Guillen LE Flores-Mir C. Björk-Jarabak cephalometric analysis on CBCT synthesized cephalograms with different dentofacial sagittal skeletal patterns. Dental Press J Orthod. (2014) 19:46–53. 10.1590/2176-9451.19.6.046-053.oar 25628079 PMC4347410

[38] Jordan AS White DP. Pharyngeal motor control and the pathogenesis of obstructive sleep apnea. Respir Physiol Neurobiol. (2008) 160:1–7. 10.1016/j.resp.2007.07.009 17869188 PMC2705920

[39] Kohno A Kitamura Y Kato S Imai H Masuda Y Sato Y Displacement of the hyoid bone by muscle paralysis and lung volume increase: the effects of obesity and obstructive sleep apnea. Sleep. (2019) (1):42. 10.1093/sleep/zsy198 PMC6335873 30371885

[40] Oka N Habumugisha J Nakamura M Kataoka T Fujisawa A Kawanabe N Exploring the relationship between posture-dependent airway assessment in orthodontics: insights from kinetic MRI, cephalometric data, and three-dimensional MRI analysis. BMC Oral Health. (2025) 25:745. 10.1186/s12903-025-06088-x 40399950 PMC12093671

[41] Hartfield PJ Janczy J Sharma A Newsome HA Sparapani RA Rhee JS Anatomical determinants of upper airway collapsibility in obstructive sleep apnea: a systematic review and meta-analysis. Sleep Med Rev. (2023) 68:101741. 10.1016/j.smrv.2022.101741 36634409 PMC11493082

[42] Xu Y Yu M Huang X Wang G Wang H Zhang F Differences in salivary microbiome among children with tonsillar hypertrophy and/or adenoid hypertrophy. mSystems. (2024) 9:e00968–24. 10.1128/msystems.00968-24 39287377 PMC11494981

